# Prognostic factors of second primary contralateral breast cancer in early-stage breast cancer

**DOI:** 10.3892/ol.2014.2623

**Published:** 2014-10-17

**Authors:** ZHENG LI, FABRICE SERGENT, MICHEL BOLLA, YUNFENG ZHOU, ISABELLE GABELLE-FLANDIN

**Affiliations:** 1 Department of Radiation and Medical Oncology, Zhongnan Hospital Affiliated to Wuhan University, Wuhan, Hubei 430071, P.R. China; 2 Department of Gynecology, Central Hospital of Grenoble University, Grenoble 38043, France; 3 Department of Radiation Oncology, Central Hospital of Grenoble University, Grenoble 38043, France

**Keywords:** breast cancer, radiotherapy, contralateral breast cancer, risk factors

## Abstract

The aim of the present study was to investigate the therapeutic outcome of early-stage breast cancer (pT1aN0M0) and to identify prognostic factors for secondary primary contralateral breast cancer (CBC). A total of 85 patients with mammary carcinomas were included. All patients had undergone breast surgery and adjuvant treatment between January 2001 and December 2008 at the Central Hospital of Grenoble University (Grenoble, France). The primary end-points were disease-free survival and secondary CBC, and the potential prognostic factors were investigated. During a median follow-up of 60 months, 10 of the 85 patients presented with secondary primary cancer, of which six suffered with CBC. No patient mortalities were reported. The rates of CBC were 2.35, 3.53 and 7.06% at one, two and five years, respectively. The cumulative univariate analysis showed that microinvasion and family history are potential risk factors for newly CBC. The current study also demonstrated that secondary CBC was more likely to occur in patients with microinvasion or a family history of hte dise. In addition, the systematic treatment of secondary CBC should include hormone therapy.

## Introduction

Breast cancer is the most common type of cancer in females in developed and developing countries. Since 2004, a French national screening program of breast cancer has been established for females between 50–74 years old (1). Mass screening for breast cancer has led to the identification of 14,500 novel breast cancers, with a rate of 6.7 cancers identified per 1,000 females screened. In 2011, breast cancer was the most common type of cancer among French females, with 53,000 new cases identified, followed by colorectal cancer (19,000 cases) and lung cancer (12,000 cases). In addition, breast cancer continued to be the leading cause of cancer-related mortality, with 11,500 mortalities, in 2011. However, the rate of breast cancer-related mortality has actually decreased since 1946 (2).

Various studies have identified a number of risk factors, including age, lymphovascular emboli or invasion (LVI), menopause, hormone receptors and the type of treatments, which may affect the survival of early-stage breast cancer patients (3–11).

Few studies has described early breast cancer and therefore, the aim of the present study was to explore whether other factors impact the survival and development of contralateral breast cancer (CBC) at the early stage.

## Materials and methods

### Patients

This study retrospectively collected the clinical and pathological data of a total of 85 early-stage breast cancer patients (pT1aN0M0) who were treated at the Central Hospital of Grenoble University (Grenoble, France) between January 2001 and December 2008. All patients underwent surgery, however, the post-operative treatments varied, including radiotherapy (RT), hormone therapy, chemotherapy and observation. The follow-up time ranged between three and 127 months. Overall, eight patients (9%) were lost to follow-up. The pre-operative examinations included annual breast cancer screening reports, taking a family history, a physical examination, routine laboratory tests, tumor marker analysis, bilateral mammography or chest radiograph, examination of the sentinel lymph node, abdominal/pelvic contrast-enhanced computed tomography and magnetic resonance imaging. This study was approved by the ethics committee of the Central Hospital of Grenoble University.

### Inclusion and exclusion criteria

The criteria used to select the patients were as follows: i) The patient must be diagnosed in the region of Rhone-Alpes and treated at the Central Hospital of Grenoble, with no history of breast cancer prior to January 2001. ii) an Eastern Cooperative Oncology Group performance status of 0–2 prior to surgery; iii) patients were included if they presented with other types of systemic disease, including hypertension and diabetes, but excluded if the condition was considered a contraindication of surgery; iv) patients did not present with other primary tumors; v) all tumors were ≤5 mm at the greatest dimension according to the pathological report; and vi) microinvasion of the primary tumor of 1–3 mm in the longest diameter, determined as ductal carcinoma *in situ* (DCIS) or lobular carcinoma *in situ* (LCIS), according to the pathological reports, and not as a satellite lesion or metastasis.

### Treatments

The treatment strategies predominantly included surgery, RT, hormone therapy, chemotherapy and observation. All patients underwent surgery, including quadrantectomy, mastectomy or lumpectomy. Examination of the lymph nodes included an examination of the sentinel lymph node and dissection of the axillary lymph nodes. In addition, all patients received RT (50 Gy/25 fractions for five weeks), with the exception of patients with microinvasion or without any trace of the tumor bed following biopsy. The patients received 50 Gy of internal mammary chain RT if the tumor was located in the internal quadrant of the breast. The administration of 45 Gy to the supraclavicular area following axillary dissection was insufficient. Either 6-MV photon X or electrons were used to administer a boost of 10 Gy to the tumor bed in patients with high-risk factors of relapse, including an age of <60 years old, R1 (it has been observed that tumor cells remain in the surgical margin when viewed under the microscope) and high-risk family history (≥1 family members have breast cancer). Patients with Her-2(+++) overexpression, according to the ASCO-CAP HER2 Test Guideline Recommendations (12) where Her-2(+++) is defined as uniform intense membrane staining of >30% of the invasive tumor cells, were administered six 21 day cycles of a fluorouracil (intravenous, 500 mg/m^2^, days 1 and 8), epirubicin (75 mg/m^2^, day 1) and cyclophosphamide (500 mg/m^2^, day 1) regimen of chemotherapy, which lasted 4.5 months. Hormone therapy with tamoxifen or anti-aromatase inhibitors was offered for hormone receptor-positive patients. Observation was only recommended for the following patients: i) Those at low-risk of recurrence, including those ≥60 years old, those with no relevant family history and a performance state of 0; and ii) those refusing any treatment following surgery. The medical characteristics of the patients are presented in Table I.

### Follow-up

Every three months, the patients were followed up and obtained results from mammography and biochemistry analyses. In total, 30% of patients returned to the Central Hospital of Grenoble University, while 61% visited their family doctors. The period of follow-up was from the date of surgery to the October 15, 2011. Local recurrence or new breast cancer were confirmed by histological examination.

### Statistical analysis

Data are presented as the mean ± standard deviation or as n (%). To assess the differences between the groups, Student’s t-test was used for continuous variables, and the χ^2^ test was used for categorical variables. P<0.05 was considered to indicate a statistically significant difference. Statistical analysis was performed using STATA software (version 10; Stata Corporation, College Station, TX, USA). Disease-free survival (DFS) time was estimated using Kaplan-Meier analysis. The log-rank test was used to evaluate the effects of different individual variable factors on the relapse-free survival time. The overall survival (OS) time was defined as the elapsed interval between the date of the initial surgery to mortality, loss to follow-up or October 15, 2011. The DFS time was defined as the time from the date of surgery to the date of local recurrence or new CBC.

## Results

### Treatment

In total, 85 patients underwent surgery and 72 patients received RT, of which, 40 were administered boost irradiation. Furthermore, 21 patients received hormone therapy and two patients received chemotherapy, which was followed by an additional herceptin treatment for one year in one patient. Herceptin dosages were dependent on the weight of the patient: herceptin dose for the first cycle (mg/kg) = 6 mg × weight of patient (kg); herceptin dosage for the second to final cycle (mg/kg) = 4 mg × weight of the patient (kg), each cycle lasts for 21 days and the overall treatment lasts for a year. Seven patients underwent observation only. No local recurrence or mortalities were observed during the follow-up period, however, 11 secondary cancers were identified in 10 patients. This consisted of five cases of secondary CBC and one each of thyroid, bladder, tongue and colon cancer. One patient was identified with cervical and breast cancer on the contralateral side.

### OS and DFS analysis

A complete follow-up was achieved in 91% (n=77) of patients, while 9% (n=8) were lost to follow-up. The follow-up period varied between three and 127 months. The median follow-up period was 60 months. No mortalities occurred during the study period. The corresponding rates of DFS by variable prognostic factors are shown in Table II.

### Prognostic factors for DFS (univariate analysis)

**Figure 1:**
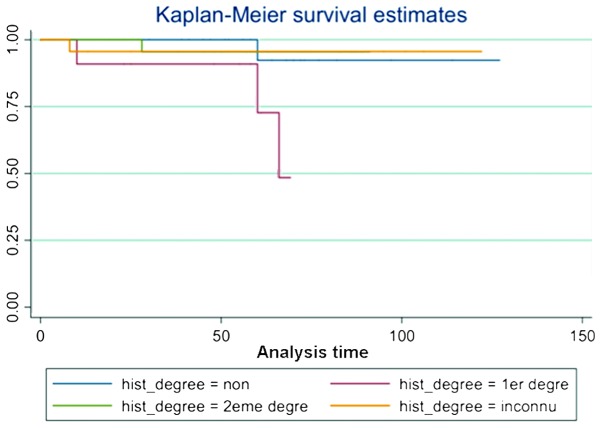
Disease-free survival by family history (Kaplan-Meier). The Kaplan-Meier estimates revealed that the first degree of family history, which describes the family member with breast cancer (daughter, mother or sibling), is the most important for the occurrence of new contralateral breast cancer. Whilst the second degree of family history (aunt or niece), without family history or other status, lessens the revolution. Hist_degree, degree of family history; non, no family history; 1ere degree, first degree family history; 2eme degree, second degree family history; inconnu, unknown.

**Figure 2:**
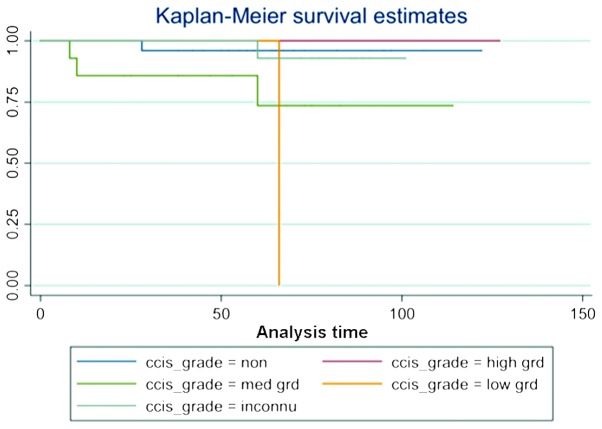
Disease-free survival by satellite lesions presented as ccis associate (Kaplan-Meier). The Kaplan-Meier survival estimates revealed that the lowest degree was considered to be the most important reason for a new occurrence. Among the patients who were detected to have low-grade ductal breast satellite lesions, only one was administered hormone therapy. ccis_grade; grade of microinvasion; non, no micorinvasion; inconnu, unknown.

As no mortalities were observed in this study, the appearance of CBC was regarded as the evolution of primary breast cancer. The cumulative recurrence for DFS was univariately affected by the known parameters of family history and microinvasion (summarized in Table III and Figs. 1 and 2).

### Patients with secondary CBC

In this study, six patients were identified with a secondary primary CBC. The patient characteristics are presented in Table IV. Only one patient was >55 years old and four had a relevant family history, with three being due to first degree relatives. The rate of CBC was 2.35, 3.53 and 7.06% at one, two and five years, respectively. Of the six females, five were menopausal, four were identified with invasive ductal carcinoma at the first diagnosis, two with invasive lobular carcinoma, five with low-grade tumors (I+II) and only one with high-grade tumors (III) according to the Bloom-Richardson grading system. By contrast, the histology of the contralateral tumor was reversed; four patients exhibited ILC, while two exhibited IDC. All of these patients received RT, however, none received hormone therapy due to a number of personal reasons. In addition, no necrosis or LVI was identified.

## Discussion

As all patients in the present study were diagnosed with early-stage breast cancer, the study aimed to understand why the rates of CBC remained so high.

CBC is considered to be the most common type of secondary cancer for those whose primary cancers are located in the breast, accounting for almost half of all secondary tumors (10). Therefore, the analysis of CBC is becoming an important public issue. The overall incidence rates of CBC vary between 4 and 8 per 1,000 individuals per year, with different stages and treatment strategies (11). With regard to the incidence of secondary CBC of early-stage breast cancer, Gao *et al* (13) observed that the rates of CBC were 2.9, 6.1, 9.1 and 12% at five, 10, 15 and 20 years, respectively. Comparatively, the incidences of 2.35, 3.53 and 7.06% at one, two and five years that were identified in the current study were marginally lower.

Among the CBC patients in the present study, five out of six were <55 years old. Although the study did not report that age impacts the rates of CBC, a number of studies have revealed that females of a young age suffer a greater risk of secondary primary breast cancer. Broët *et al* (14) identified that patients <55 years old [relative risk (RR), 1.40; 95% confidence interval (CI), 1.10–1.78] were associated with an increased risk of CBC. However, another study considered an age of ≤45 years to be a risk factor (15). By contrast, compared with the ages of between 45 and 55 years, Gao *et al* (13) found an age of >55 years to be a risk factor.

In the present study, family history was a key potential risk factor of CBC; this has been confirmed by a number of studies. Reiner *et al* (16) considered females of <45 years old with first degree relatives to be at the highest risk (RR, 2.5; 95% CI, 1.1–5.3). A study by Yadav *et al* (17) also showed that females with a family history had the highest incidence rates of CBC (15.3%; RR, 1.6; 95% CI, 1.12–1.27) at 20 years old. Additionally Lizarraga *et al* (18) found that having multiple first and second degree relatives appeared to increase the risk of CBC by two- or three-fold.

The impact of microinvasion on the rate of CBC remains controversial, and has been investigated by few studies (19). It is not easy to determine whether microinvasion is a risk factor for CBC or metastasis. However, in this study, the statistical analyses revealed that microinvasion does impact the rates of CBC. A study by Claus *et al* (20) demonstrated that patients whose primary tumor was diagnosed as LCIS were 2.6 times (95% CI, 2.0–3.4%) more likely to develop CBC within the first six months of the initial primary tumor compared with females with DCIS. If the period of follow-up can be extended or more early-stage breast cancer patients are excluded, the potential effect of microinvasion may be observed.

As shown in Table IV, none of the CBC patients received hormone therapy, however, all the patients exhibited indicators of suitability for hormone therapy according to their positive status of ER/PR, which markedly increases the incidence of CBC. Tamoxifen, as a representative of hormone therapy, is well known to reduce the risk of CBC (21,22). Furthermore, in the latest large multiple center study (23), 1,583 patients with BRCA1 mutations and 881 with BRCA2 mutations, 383 (24%) and 454 (52%) of patients were administered tamoxifen, respectively, following the initial breast cancer diagnosis. This cohort study revealed that the use of tamoxifen may reduce the risk of CBC for BRCA1 and BRCA2 mutation carriers. However, a study among elderly patients (≥65 years old) who were classified as T1N0M0 and treated with breast-conserving surgery and RT showed no significant differences in the 10-year survival of CBC patients or OS between the tamoxifen and non-tamoxifen cohorts (24).

The serine protease urokinase-type plasminogen activator (uPA) and its inhibitor, PAI-1, are considered to be independent, statistically prognostic factors in primary breast cancer. The NNCB-3 trial (23) confirmed the highest level of evidence for the clinical utility of uPA and PAI-1. Furthermore, uPA and PAI-1 also serve as predictive factors of response to adjuvant therapy and the early relapse of breast cancer. At present, examination of UPA/PAI-1 has been essential in the pathological surgical studies of breast cancer in France (25–30), and may be investigated in our future retrospective studies.

In the present study, five other secondary primary cancers were identified, including thyroid, bladder, tongue, colon and cervical cancer. It is likely that multiple factors, including genetic effects, endogenous hormones, pollution, environmental exposure, age and the initial treatments for primary breast cancer, resulted in variations between the standardized incidence ratios in cancer of the digestive system, lungs, uterus, ovaries, kidneys and bladder, soft tissue sarcoma, melanoma and certain types of hematological malignancy (31,32).

In the current study, family history and microinvasion were poor prognostic factors. The most likely reason for this result was insufficient systemic treatment, particularly from hormone therapy. At present, although a consensus has not been reached on the use of adjuvant treatment for early-stage breast cancer, a large quantity of observational and follow-up examinations are being conducted by family doctors, and regularly communication between family doctors and oncologists should be encouraged and regarded as a routine procedure. Oncologists or family doctors should persuade the patients who exhibit indicators of suitability for hormone therapy (positive ER/PR status) to continue the treatment. Furthermore, an increased period of follow-up must be implemented and other significant biomarkers investigated to continue this study further.

## Figures and Tables

**Table I:** Patient characteristics (n=85).

Characteristics	n
Age, years	
≤55	37
>55	48
Postmenopausal	
Yes	55
No	22
Unknown	8
Family history	
1st degree	12
2nd degree	24
No	26
Unknown	23
Surgery type	
Lumpectomy	2
Mastectomy	33
Qyadrantectomy	50
Histology type	
ILC	12
IDC	71
Others	2
SBR	
Grade I	37
Grade II	23
Grade III	9
Unknown	16
Microinvasion	
High-grade	16
Medium-grade	15
Low-grade	3
None	26
Unknown	25
Adjuvant treatment	
HT	3
HT+RT	17
RT	54
CT+RT	1
CT+HT	1
Observation	9
Hormone receptor	
ER^+^/PR^+^	50
ER^−^/PR^−^	7
ER^+^/PR^−^	19
ER^−^/PR^+^	5
Unknown	4
Boost technique	
Yes	45
No	40
Her-2(+++)	
Yes	9
No	39
Unknown	37

HT, hormonotherapy; RT, radiotherapy; CT, chemotherapy; ILC, invasive lobular carcinoma; IDC, invasive ductal carcinoma; ER, estrogen receptor; PR, progesterone receptor; MBRs, membrane bioreactors; SBR, Scarff-Bloom-Richardson.

**Table II:** DFS by variable prognostic factors (n=85).

	DFS, %			
	
Factor	n	1-year (95% CI)	3-year (95% CI)	5-year (95% CI)
Age, years				
≤55	37	97.2 (81.8–99.6)	97.2 (81.8–99.6)	87.0 (63.7–95.8)
>55	48	97.9 (85.8–99.7)	95.6 (83.5–98.9)	95.6 (83.5–98.9)
Postmenopausal				
Yes	55	98.2 (87.6–99.7)	96.1 (85.2–99.0)	92.4 (77.0–97.7)
No	22	95.2 (70.7–99.3)	95.2 (70.7–99.3)	95.2 (70.7–99.3)
Unknown	8	100.0	100.0	83.3 (27.3–97.5)
Family history				
1st degree	12	91.0 (50.8–98.7)	91.0 (50.8–98.7)	72.7 (24.1–93.1)
2nd degree	24	100.0	95.5 (71.9–99.3)	95.5 (71.9–99.3)
No	26	100.0	100.0	92.3 (56.6–98.9)
Unknown	23	95.7 (72.9–99.4)	95.7 (72.9–99.4)	95.7 (72.9–99.4)
Surgery type				
Lumpectomy	2	100.0	100.0	100.0
Mastectomy	33	96.9 (79.8–99.6)	96.9 (79.8–99.6)	92.0 (70.8–98.0)
Quadrantectomy	50	98.0 (86.4–99.7)	95.7 (83.8–98.9)	91.1 (72.9–97.3)
Histology type				
ILC	12	100.0	90.9 (50.8–98.7)	90.9 (50.8–98.7)
IDC	71	97.1 (88.9+99.3)	97.1 (88.9+99.3)	91.7 (79.5+97.0)
Others	2	100.0	100.0	100.0
SBR				
I	37	97.2 (81.9–99.6)	97.2 (81.9–99.6)	90 (71.9–96.7)
II	23	100.0	94.7 (68.1–99.2)	94.7 (68.1–99.2)
III	9	88.9 (44.3–98.4)	88.9 (44.3–98.4)	88.9 (44.3–98.4)
Unknown	16	100.0	100.0	100.0
Microinvasion				
High-grade	16	100.0	100.0	100.0
Medium-grade	15	85.7 (53.9–96.2)	85.7 (53.9–96.2)	73.5 (35.9–91.1)
Low-grade	3	100.0	100.0	0.0
None	26	100.0	96.0 (74.8–99.4)	96 (74.8–99.4)
Unknown	25	100.0	100.0	92.9 (59.1–99.0)
Adjuvant treatment				
HT	3	100.0	100.0	100.0
HT+RT	17	100.0	100.0	100.0
RT	54	98.1 (87.4–99.7)	96.1 (85.3–99.0)	89.7 (74.1–96.1)
CT	1	100.0	100.0	100.0
CT+HT	1	100.0	100.0	100.0
Observation	9	88.9 (43.3–98.4)	88.9 (43.3–98.4)	44.4 (1.0–86.6)
Hormone receptor				
ER^+^/PR^+^	50	97.9 (86.1–99.7)	97.9 (86.1–99.7)	89.4 (69.3–96.6)
ER^+^/PR^−^	19	100.0	94.1 (65.0–99.2)	94.1 (65.0–99.2)
ER^−^/PR^−^	7	100.0	100.0	100.0
ER^−^/PR^+^	5	80.0 (20.4–96.9)	80.0 (20.4–96.9)	80.0 (20.4–96.9)
Unknown	4	100.0	100.0	100.0
Boost technique				
Yes	45	100.0	97.1±2.9	91±6.5
No	40	95.6±3.1	95.6±3.1	92±4.6
Her-2(+++)				
Yes	9	100.0	100.0	100.0
No	39	94.9 (81.0–98.7)	94.9 (81.0–98.7)	88.6 (65.0–96.7)
Unknown	37	100.0	97.0 (80.3–99.6)	93.2 (75.4–98.3)

DFS, disease-free survival; HT, hormone therapy; RT, radiotherapy; CT, chemotherapy; ILC, invasive lobular carcinoma; IDC, invasive ductal carcinoma; ER, estrogen receptor; PR, progesterone receptor; MBRs, membrane bioreactors; SBR, Scarff-Bloom-Richardson; CI, confidence interval.

**Table III:** Univariate analysis by multiple potential factors for DFS (log-rank test).

Factor	n	P-value
Age, years		
≤55	37	
>55	48	0.6006
Postmenopausal		
Yes	55	
No	22	
Unknown	8	0.8589
Family history		
1st degree	12	
2nd degree	24	
No	26	
Unknown	23	0.0352^a^
Surgery type		
Lumpectomy	2	
Mastectomy	33	
Quadrantectomy	50	0.7785
Histology type		
ILC	12	
IDC	71	
Others	2	0.9317
SBR		
I	37	
II	23	
III	9	
Unknown	16	0.5814
Microinvasion		
High-grade	16	
Medium-grade	15	
Low-grade	3	
None	26	
Unknown	25	0.0425^a^
Adjuvant treatment		
HT	3	
HT+RT	17	
RT	54	
CT	1	
CT+HT	1	
Observation	9	0.1916
Hormone receptor		
ER^+^/PR^+^	50	
ER^−^/PR^−^	7	
ER^+^/PR^−^	19	
ER^−^/PR^+^	5	
Unknown	4	0.6019
Boost technique		
Yes	45	
No	40	0.6546
Her-2(+++)		
Yes	9	
No	39	
Unknown	37	0.3722

a P<0.05.

DFS, disease-free survival; HT, hormone therapy; RT, radiotherapy; CT, chemotherapy; ILC, invasive lobular carcinoma; IDC, invasive ductal carcinoma; ER, estrogen receptor; PR, progesterone receptor; MBRs, membrane bioreactors; SBR, Scarff-Bloom-Richardson.

**Table IV:** Characteristics of patients with new contralateral breast cancer.

Patients	1	2	3	4	5	6
Age, years	47	74	50	53	52	50
Family history, degree	1st	2nd	1st	No	No	1st
Interval of new tumor, years^a^	1	2	5	5	1	5
Menopausal	No	Yes	Yes	Yes	Yes	Yes
ER/PR	+/−	+/+	+/+	+/+	+/+	+/+
Primary histology	IDC	ILC	ILC	IDC	IDC	IDC
Primary histology grade	III	II	I	I	I	I
Contralateral cancer histology	IDC	ILC	ILC	ILC	IDC	ILC
Size of the second tumor, mm	Unknown	Unknown	26	9	7	26
Initial surgery	MT	QT	QT	QT	QT	MT
Initial RT	No	Yes	Yes	Yes	Yes	Yes
Initial HT	No	No	No	No	No	No
Necrosis/LVI	No	No	No	No	No	No
Microinvasion, grade	Medium	No	Low	Medium	Medium	Unknown
First margin of surgery	(−)	(−)	(−)	(−)	(−)	(−)

a Interval between the time of treatment for the primary breast cancer to the diagnosis of the new cancer.

IDC, invasive ductal carcinoma; ILC, invasive ductal carcinoma; QT, quadrantectomy; MT, mastectomy; LVI, lymphovascular emboli or invasion; ER, estrogen receptor; PR, progesterone receptor; HT, hormonotherapy; RT, radiotherapy.
